# A novel assay for analysis of the regulation of the function of human osteoclasts

**DOI:** 10.1186/1479-5876-4-45

**Published:** 2006-11-07

**Authors:** Barrie Kirstein, Urszula Grabowska, Bertil Samuelsson, Masahiro Shiroo, Timothy J Chambers, Karen Fuller

**Affiliations:** 1 Department of Cellular Pathology, St George's, University of London, London SW17 0RE, UK; 2 Medivir UK, Little Chesterford, Essex CB10 1XL, UK

## Abstract

**Background:**

Very little is known of the regulation of the function of human osteoclasts, largely due to the virtual impossibility of obtaining human osteoclasts *ex vivo*. It has recently become possible to generate human osteoclasts *in vitro*, by incubation of peripheral blood mononuclear cells (PBMCs) in macrophage colony-stimulating factor (M-CSF) and receptor activator of nuclear factor-κB ligand (RANKL). However, the assays at present available do not distinguish clearly between the distinct effects of agents on differentiation and function.

**Materials and methods:**

We developed a novel assay for resorptive function of human osteoclasts that minimizes inter-assay variability by using each culture as its own baseline, and that minimizes the confounding effects of agents on differentiation by assessing resorptive function over a short test period. In this assay, the development of resorptive activity is monitored in sample cultures. When resorption is underway, bone resorption (measured as the release of the C-terminal telopeptide degradation product of type I collagen (CTX-I) into the supernatant) is compared before *vs *after incubation for 1–24 h in test agent.

**Results:**

Using this assay, we found that changes in bone resorption could be detected using substantially fewer cultures per variable. Moreover, we could detect effects of agents on resorption within 1 h of addition, a time sufficiently short that a change in release is likely to reflect an effect on function rather than on differentiation.

**Conclusion:**

The assay makes it possible to distinguish the effects of agents on osteoclastic function, independent of their effects on differentiation.

## Background

The maintenance of skeletal integrity depends on continual resorption of bone by osteoclasts and its replacement by osteoblasts. Recently, there have been considerable advances in our understanding of the mechanisms through which osteoclast formation is regulated [1-3]. In contrast, little is known of the mechanisms that modulate their activity once formed, even although this is a major component of the regulation of bone resorption. Thus, after systemic administration of hormones such as PTH or CT, osteoclasts show morphological evidence of an increase or decrease in activity, with a corresponding change in plasma calcium concentration, within 30 min, while a change in osteoclast number is not detectable until 24 h later [4]. This shows that bone resorption is regulated not only through modulation of the number of osteoclasts but also by modulation of the resorptive activity of existing osteoclasts. It seems likely that agents exert differential actions on these distinct processes.

It is virtually impossible to obtain human osteoclasts *ex vivo *with which to address this question. In their absence, human osteoclastic cells can be generated *in vitro *by incubation of peripheral blood mononuclear cells (PBMCs) in macrophage colony-stimulating factor (M-CSF) and receptor activator of nuclear factor-kB ligand (RANKL) on plastic culture surfaces or on bone/dentine slices [5,6]. However, although such culture systems provide powerful insights into the regulation of osteoclastic differentiation, they do not clearly distinguish between the effects of agents on differentiation and function. For example, resorption of bone slices in such culture systems is typically observed after 14–21 days of incubation [7-10]. If a putative resorption modulator is added to such cultures for a brief period, effects on resorption, classically measured as the area of bone surface excavated, will be observed against a baseline of prior resorption; and if the modulator is added over a longer period, it will be difficult to distinguish effects on function from those on differentiation.

Recently, it has become possible to measure bone resorption as the release into culture supernatants of products of bone solubilization, the concentration of which has been shown to correlate with bone resorption [11]. This approach has the advantage that the amount released reflects the amount of bone resorbed since the last change of culture medium. This avoids the results being masked by the baseline of prior resorption. However, even in such assays, bone resorption is measured over a period of 3 days [12,13], so that an unknown and potentially substantial component of an observed change in resorptive activity might have been due to an effect of the test agent on differentiation rather than function.

The difficulties in the interpretation of resorption data mentioned above are compounded by the length of time it normally takes for osteoclastic differentiation to occur: the long incubation times magnify small initial differences between cultures, and in our experience can lead to substantial inter-culture variability. This variability increases the number of cultures required per variable, and because relatively small numbers of monocytes are available from a given donor, the number of variables that can be studied in each experiment is in practice severely limited.

We therefore developed a novel assay that minimizes the confounding effects of agents on differentiation by measuring resorption over a shorter period; and that minimizes inter-culture variability by using each culture as its own baseline. In this assay, sample cultures are inspected to monitor the development of actively resorbing osteoclasts. When resorption is underway, release of products of bone resorption is compared before *vs *after incubation for 1–24 h with test agent. Using this approach, we found that changes in bone resorption could be detected using substantially fewer cultures per variable; and that effects of agents on resorption could be detected within 1 h of addition, a time sufficiently short that a change in release is likely to reflect an effect on function rather than on differentiation.

## Materials and methods

### Media and reagents

Cells were incubated in MEM with Earle's salts (Invitrogen, Paisley, UK), supplemented with 10% fetal calf serum (FCS), 2 mM glutamine, 100 IU/ml benzylpenicillin, and 100 μg streptomycin (all Sigma, Poole, Dorset, UK) (MEM/FCS). Recombinant human M-CSF and soluble recombinant murine RANKL were from Insight Biotechnology (Wembley, Middlesex, UK). The specific cathepsin K inhibitors MV061194, MV061748, MV061940, MV061645 and MSX-081 were provided by Medivir UK (Cambridge, UK). Cortical bovine bone slices (4 × 4 × 0.1 mm) were prepared as previously described [14]. Salmon CT, E64 and all remaining reagents were from Sigma unless otherwise stated.

### Osteoclast generation

Heparinized blood was obtained from healthy human male or female volunteers (aged 22 – 57) with the consent of St George's Ethical Committee. The blood was layered over Histopaque-1077 and centrifuged for 30 min at 400 g. The opaque interface containing mononuclear cells (PBMCs) was collected with a pasteur pipette, washed in PBS, then resuspended in MEM/FCS. 5 × 10^5 ^cells were added per well to a 96-well plate, each well of which contained a bone slice. The cultures were incubated in a total volume of 200 μl MEM/FCS containing M-CSF (50 ng/ml) and RANKL (30 ng/ml) for 24 h at 37°C in 5% CO_2 _in humidified air. Bone slices were then removed from the wells and placed in the wells of a 24-well plate, in 1 ml of MEM/FCS containing M-CSF and RANKL at the above concentrations, before continued incubation. Cultures were fed three times per week by replacing 60% of the medium with fresh medium and cytokines.

**Figure 1:**
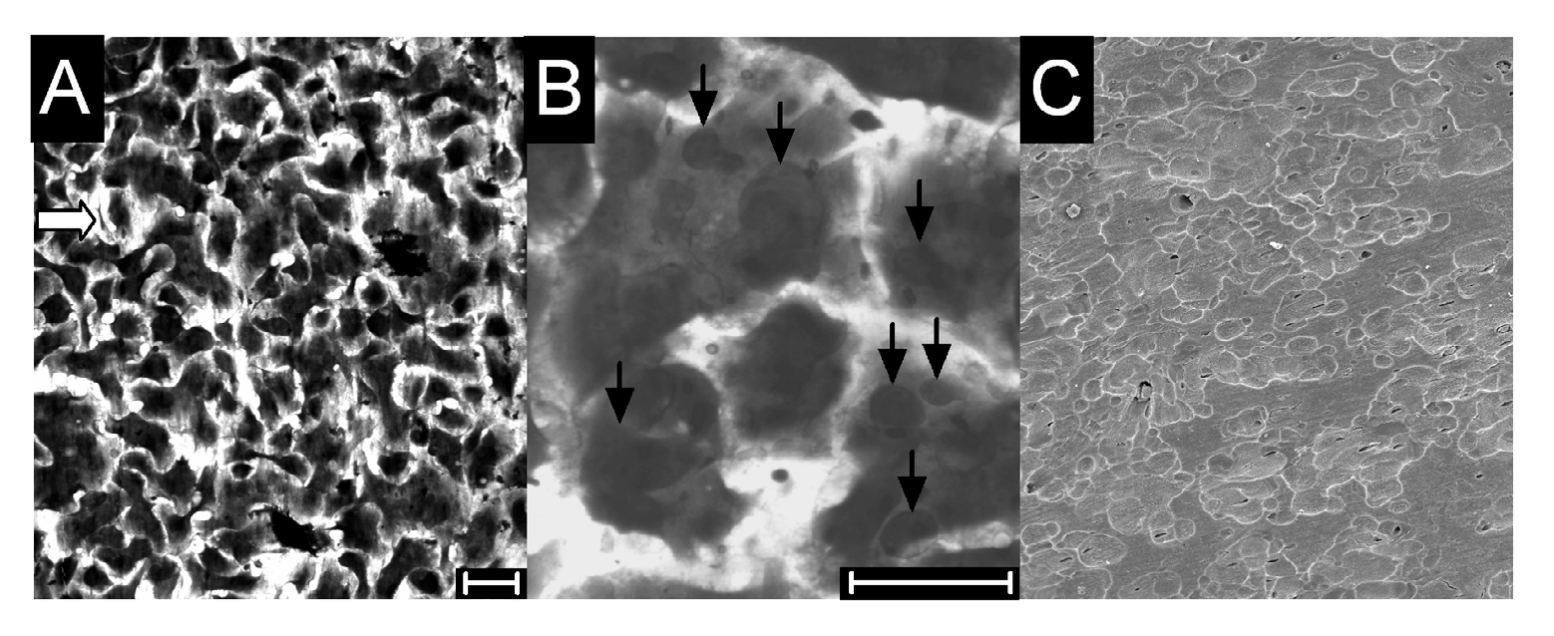
Osteoclast formation from human PBMCs. PBMCs were incubated with M-CSF and RANKL on bone slices for 7 days. Sample bone slices were taken and stained with toluidine blue (A, B) to assess multinuclear cell formation and bone resorption (bar = 100 μm). In A, virtually all of the cells shown are multinuclear, with only a very occasional cell (white arrows) remaining mononuclear. B: at higher magnification many excavations (black arrows) can be visualized as darkly-staining areas beneath the osteoclasts. C: SEM of bone slice after removal of cells. Almost the entire surface shows excavation, with only occasional islands of unresorbed surface remaining.

To monitor cultures for the development of resorptive function, sample bone slices were removed at intervals, fixed in 10% formalin and stained using toluidine blue (0.1% for 1 min). Bone slices were then inspected by light microscopy for the presence of osteoclasts and excavations (see Fig. 1). When excavation was deemed sufficient, the remaining cultures were subjected to the osteoclast resorption assay.

### Osteoclast Resorption Assay

After generation of resorptive osteoclasts (as detected above), bone slices were removed from the 24-well plates, washed in PBS, placed in the wells of a 96-well plate, and incubated for 2 or 24 h in 100 μl of fresh MEM/FCS containing M-CSF and RANKL. All the culture medium was removed after this period of incubation and stored frozen. Bone slices were washed again in PBS and transferred to new 96-well plates, the wells of which contained 100 μl of fresh MEM/FCS containing M-CSF and RANKL, together with test agent or vehicle control unless otherwise stated. Supernatants were collected and stored frozen after a further 1–24 h of incubation.

The resorption activity of the cultures was determined by quantifying the C-terminal telopeptide degradation product of type I collagen (CTX-I/CrossLaps^®^) (Nordic Bioscience Diagnostics A/S, Herlev, Denmark) in the culture supernatants. Enzyme-linked immunosorbent assay (ELISA) was performed according to the manufacturer's instructions.

In some experiments, when the resorption assay had been completed, bone slices were further analyzed for visualization of cells and excavations using toluidine blue, tartrate-resistant acid phosphatase (TRAP) cytochemistry and reflected light or scanning electron microscopy. In other experiments, the total quantity of TRAP present in the cells at the end of the culture was assessed by enzyme assay of TRAP in cell lysates.

### Reflected Light and Scanning Electron Microscopy

After incubation, bone slices were immersed in 10% (v/v) sodium hypochlorite for 10 min to remove cells. The bone slices were then washed in water, air-dried, mounted and sputter-coated with gold. Bone slices on stubs were inspected in a scanning electron microscope (S90: Cambridge Instruments, Cambridge, UK). The extent of bone resorption on bone slices mounted onto microscope slides was quantified by counting the number of grid intersections in an eyepiece graticule that overlay an area of bone resorption using reflected light microscopy.

### Enzyme Assay for TRAP

After removal of supernatant, bone slices were washed in PBS, transferred to fresh wells of a 96-well plate and incubated in 100 μl 0.1% Triton in water (v/v) for 10 min. TRAP enzyme activity was measured by the conversion of *p*-nitrophenyl phosphate to *p*-nitrophenol in the presence of sodium tartrate. 80 μl of each lysate, diluted appropriately, was added to 96-well plate wells containing 80 μl 0.09 M citrate buffer (pH 4.8) with 20 mM phosphatase substrate and 80 mM tartaric acid and incubated at room temperature for 40 min. The reaction was stopped by addition of 40 μl of 0.5 M sodium hydroxide. Optical absorbance was measured at 405 nm on an Opsys MR plate reader (Thermo Electron Corporation, Basingstoke, Hampshire, UK) against a standard curve of *p*-nitrophenol.

### Statistical Analysis

All data are expressed as mean ± standard error of mean, of four replicate cultures, unless otherwise stated. The significance of differences between control and experimental groups was assessed by Student's t test or ANOVA (Dunnett's Multiple Comparison Test). Differences were considered significant if *p *< 0.05

## Results

### Optimization of osteoclast formation and bone resorption

Previous assays have used an incubation time of three days to measure CTX-I release [12,13]. To distinguish between actions of agents on the differentiation and function of osteoclasts requires as short a sampling period as possible, and this in turn depends upon optimizing the rate of bone resorption in the cultures. The optimal density of cells for osteoclast generation from human PBMCs has been established [6,8]. It is also essential to optimize choice of donor, because there is considerable variation in the ability of PBMCs from different donors to generate osteoclasts. We also found that efficient osteoclastic differentiation was supported by only a minority of serum batches. To optimize the assay, our strategy was to test the ability of 4–5 serum batches to support osteoclast formation from the blood of 4–5 donors. This approach identified a single combination of donor and serum that efficiently generated osteoclasts. We found that the batch of serum so identified as optimal was able to similarly support efficient osteoclast formation in approximately 20% of donors; and that donors showed a reproducible ability for the efficient generation of osteoclasts. Thus, in our experience, some donors seemed able to generate only small or very small numbers of osteoclasts whatever serum batch was used; while some sera did not support osteoclast formation from any of the donors. Optimal combinations of donor and serum were thus identified that generated cultures in which virtually all cells were multinuclear, and almost the entire bone surface was excavated within 7 days (see Fig. 1)

### Optimization of resorption assay

**Figure 2:**
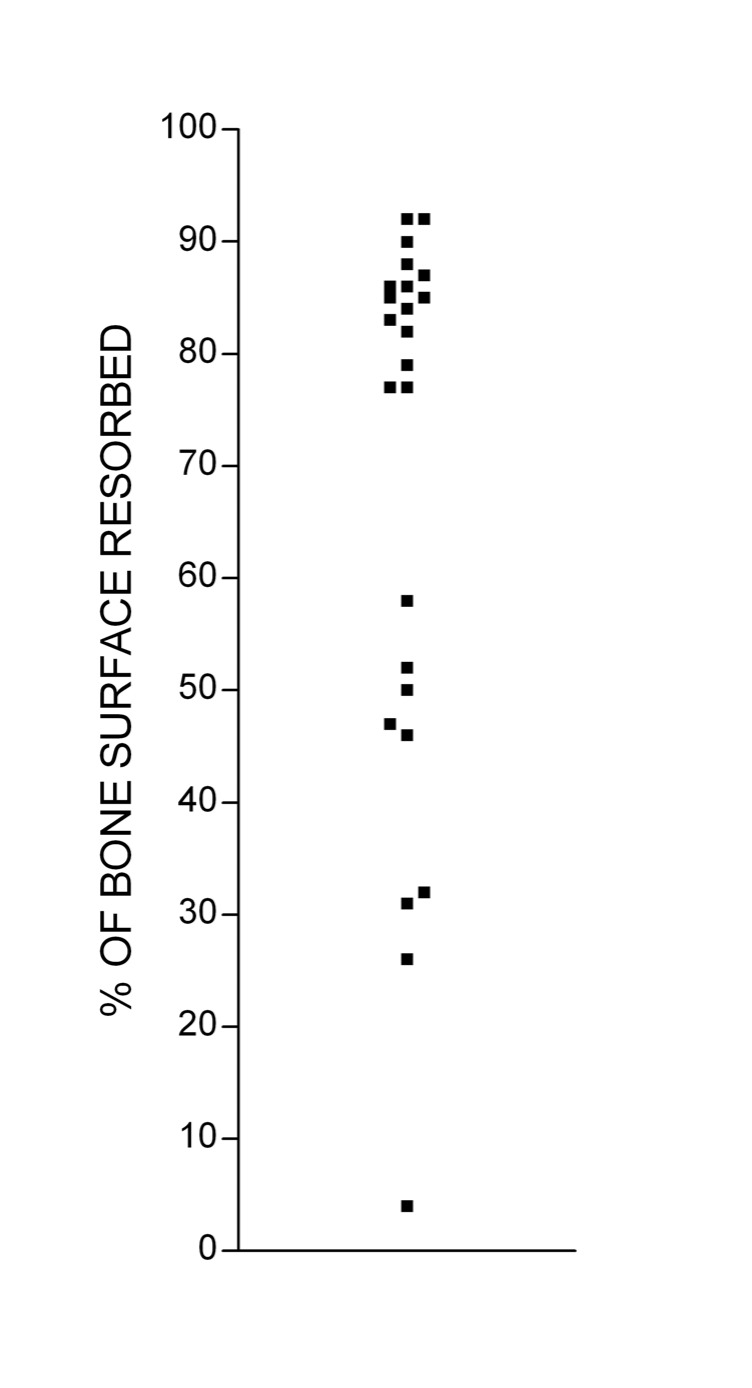
Quantification of bone resorption on individual bone slices in the same experiment. A single preparation of PBMCs was added to multiple bone slices. Bone slices were incubated in different wells with M-CSF and RANKL for 10 days. At the end of the culture period cells were removed, and the area of each bone slice excavated by osteoclasts was measured.

We noted that there was frequently considerable variation in the extent of bone resorption on different bone slices incubated under identical conditions in the same experiment (Fig. 2). This variability might reflect variation in the properties of individual bone slices. An alternative explanation is that variability might be due to the smallness of the numbers of osteoclast precursors in our cultures: these are known to represent a very small proportion (<1%) of PBMCs [15], and if the number of precursors in each culture is very small, random variation in this number could lead to appreciable inter-culture differences in osteoclast formation. A further possibility is that osteoclast formation in such cultures might reflect a cell-density dependent component in the differentiation of PBMCs to osteoclasts, which magnifies small starting differences in the number or distribution of PBMCs. Whatever the explanation for the variability, it represents an obstacle to the practical application of assays of osteoclast differentiation and function.

**Figure 3:**
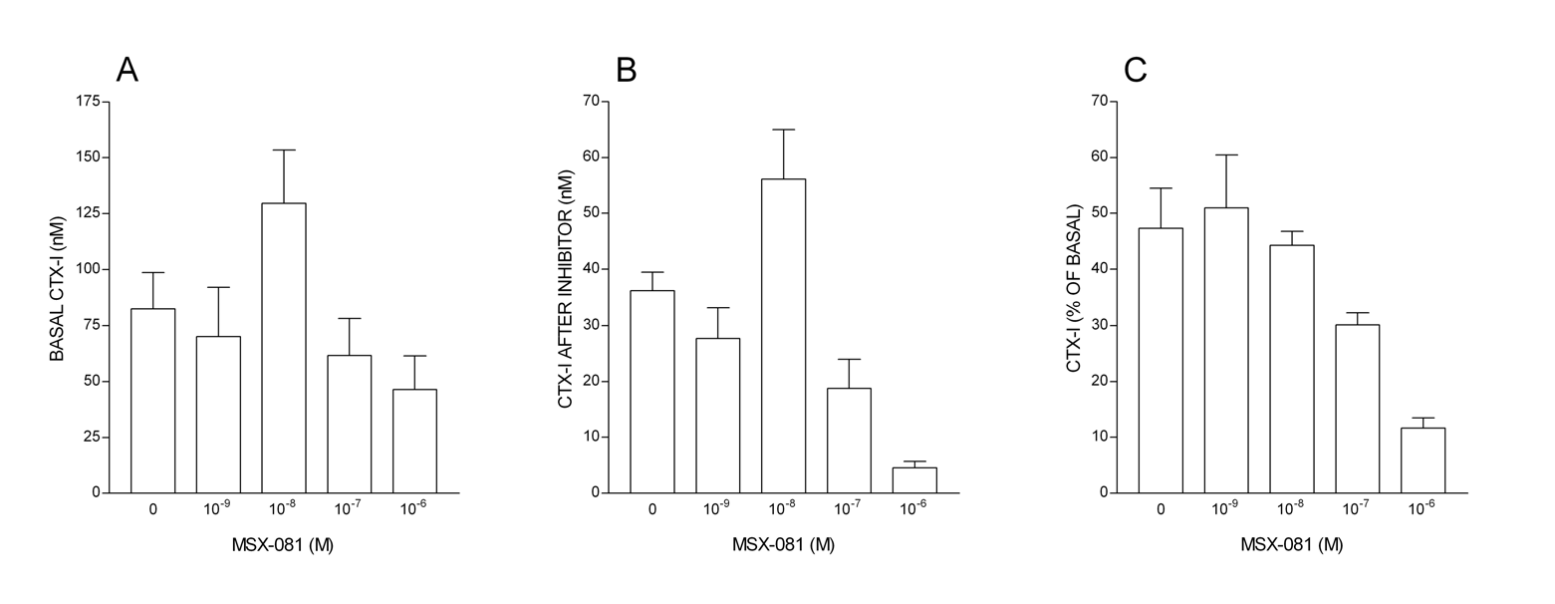
CTX-I release in the 24 h period before ('basal') (A) and after (B) addition of an inhibitor of cathepsin K, and the same data expressed (C) as CTX-I released after, as a percentage of that released in the same culture before, incubation in inhibitor. Results are derived from a single PBMC preparation. n = 4 bone slices per group.

**Figure 4:**
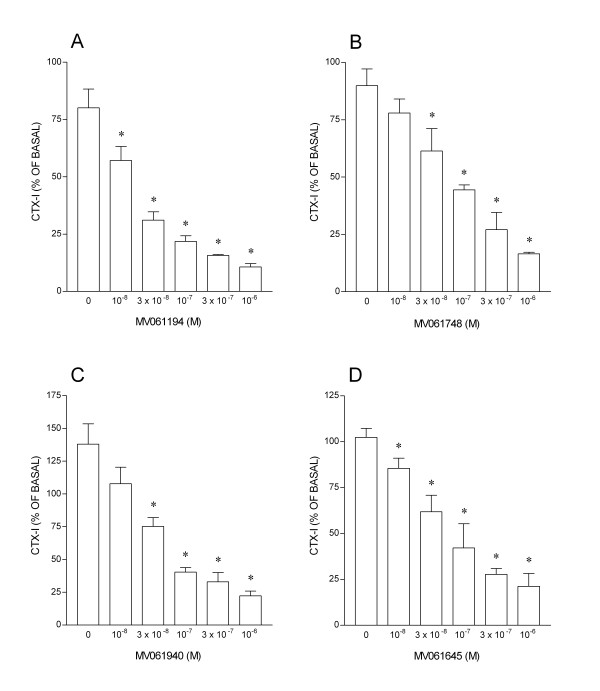
Application of assay to assessment of potency of cathepsin K inhibitors. Results expressed as CTX-I after incubation in inhibitor for 24 h, as percentage of CTX-I in the same culture measured in the 24 h before addition of the inhibitor ('basal' CTX-I release). Mean values (nM) of basal CTX-I groups were (range): A: 112–333; B: 199–379; C: 77–137; D: 185–322. n = 4 cultures per group. * p < 0.05 *vs *no inhibitor.

We hypothesized that the effect of inter-culture variability could be minimized by a longitudinal rather than cross-sectional assay design. These alternative approaches were compared in an assay using an inhibitor of cathepsin K, an enzyme crucial to the degradation of collagen during bone resorption by osteoclasts (Fig. 3). The inter-culture variability in CTX-I levels in samples taken after the initial 24 h incubation period lead to considerable variation in the means for each group (Fig. 3A). This inter-culture variability confounds the dose-response analysis derived from the CTX-I concentrations obtained after incubation in the inhibitor (Fig. 3B). However, the effect of inter-culture variability is reduced when the initial 24 h period is taken as a baseline for the second sample from the same culture (Fig. 3C). Further examples of the application of this approach to the assessment of the potency of inhibitors of bone resorption are shown in Fig. 4.

To assess the relative statistical power of the longitudinal *vs *the cross-sectional approach, we used data from the control groups of assays similar to those shown in Fig. 3. We compared the power of CTX-I data derived from the longitudinal measurements with the power of data derived from the second 24 h incubation alone. We found, in data from 6 consecutive assays, that to have a 95% chance of detecting a change in the mean of 50% required 25 (± 20) cultures if a single CTX-I measurement is used for each culture, but only 3.4 (± 2.1) cultures if cultures are sampled longitudinally.

The high cost of CTX-I assays led us to test alternative approaches that might provide a measure of the osteoclastic content of cultures that would substitute for the first CTX-I reading. It might be that the TRAP content of lysates reflects the resorptive capacity of cultures. We therefore tested the relationship between the first of the pair of CTX-I readings, and the TRAP in the lysate of the corresponding culture after incubation in either control medium or the cysteine protease inhibitor E64 (10^-7 ^M – 10^-5 ^M). We found a strong correlation between initial CTX-I and TRAP in cultures incubated in control medium (r^2 ^= 0.96) (n = 24), but the correlation was less clear for cultures incubated in E64 (r^2 ^= 0.52) (n = 24). This conforms to our previous experience, that the TRAP content of cells is influenced by agents that modulate bone resorption [16]: TRAP is released during bone resorption, and perturbation of resorption increases or decreases the amount that remains in the cell. Thus, measurement of the TRAP content of lysates is an unreliable substitute for the initial CTX-I measurement.

**Figure 5:**
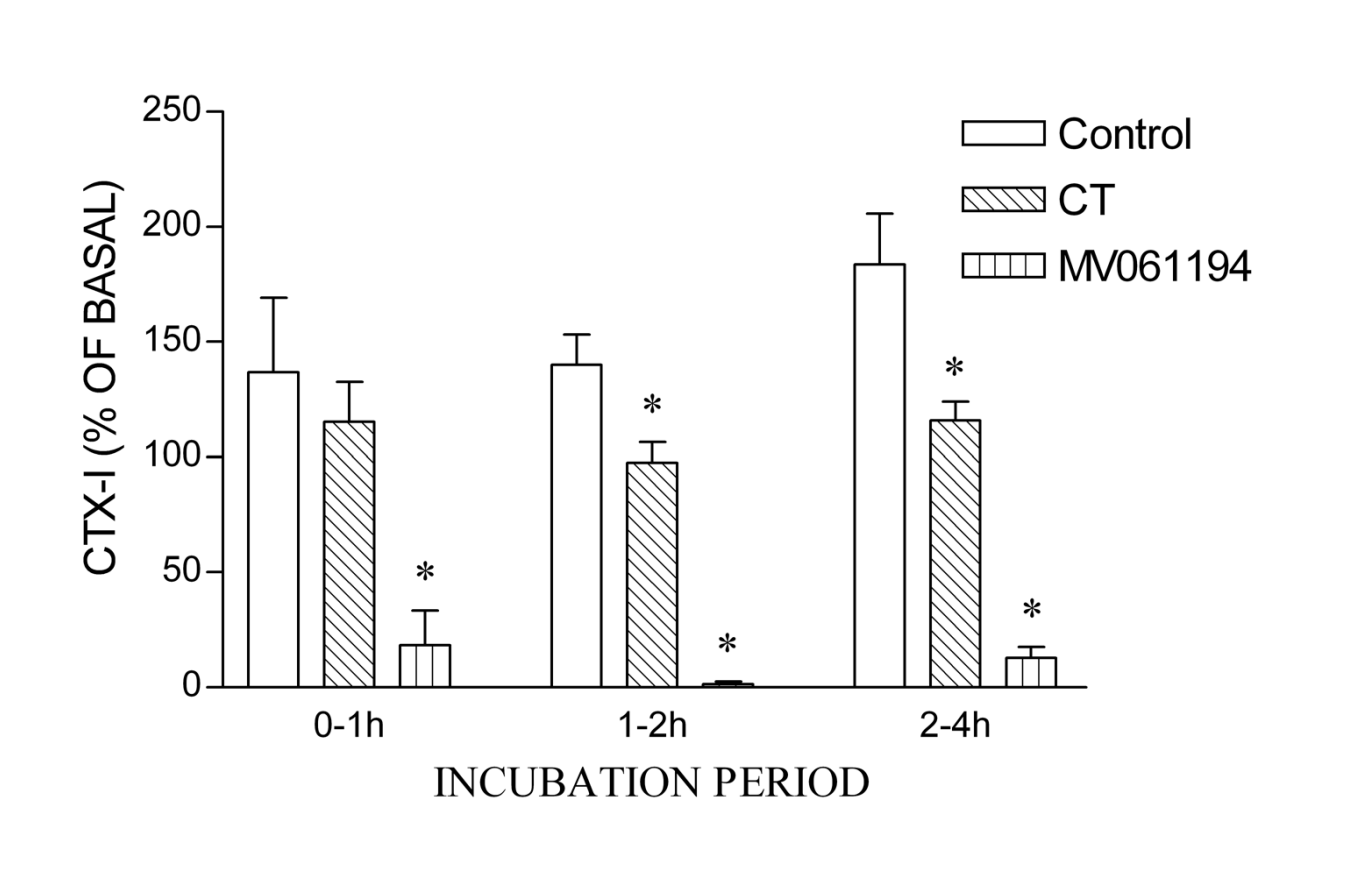
Short-term effects of resorption-inhibitors on release of CTX-I by human osteoclasts. When bone resorption was underway, as judged from inspection of sample bone slices, bone slices were washed and transferred to new wells containing fresh medium containing M-CSF/RANKL. After 2 h of incubation this supernatant was removed for CTX-I assay, and replaced with medium containing M-CSF/RANKL, together with test reagents or vehicle. Cultures were incubated for a further 1 h. This supernatant was then itself removed for assay, and the process repeated with further incubation periods of 1 and 2 h. CTX-I release was calculated as nM released per hour, and expressed as a percentage of that released per hour before the test periods. CTX-I release before test period (nM): controls: 22 ± 2.5; salmon CT (10 ng/ml): 35 ± 12; MV061194 3 × 10^-7 ^M): 34 ± 13. n = 4 cultures per group.

We tested the ability of the assay to detect changes in bone resorption over shorter time periods (Fig. 5). In these experiments, bone slices were washed after osteoclasts had formed, transferred to new 96-well plates, and incubated in fresh medium containing M-CSF and RANKL. After 2 h of incubation this supernatant was removed for CTX-I assay, and replaced with medium containing control or test reagents, and incubated for a further 1 h. This supernatant was then itself removed for assay, and the process repeated with further incubation periods of 1 and 2 h. We found that suppression of bone resorption could be detected within 1 h of incubation of osteoclasts in MV061194 (3 × 10^-7 ^M), a cathepsin K inhibitor, and within 2 h of incubation in salmon CT (10 ng/ml). This result additionally suggests that inhibition of bone resorption by the cathepsin K inhibitor has a more rapid onset than inhibition by CT. This might be because intracellular degradation of endocytosed matrix fragments continues to completion despite cell-inhibition, while the effects of the (membrane-permeant) enzyme inhibitor are essentially immediate.

## Discussion

Very shortly after administration of hormones such as CT or PTH, osteoclasts show morphological evidence *in vivo *of changes in functional activity that correspond to changes in plasma calcium concentration [4]. Only much later do osteoclast numbers change. Thus, modulation of the activity of existing osteoclasts is a major component of the regulation of bone resorption. Recently, significant advances have been made in elucidating the mechanisms that govern osteoclastogenesis [1,3,17]. In contrast, much less is known about how the resorptive activity of osteoclasts is regulated.

This is because human osteoclasts are essentially unavailable *ex vivo*. In their absence, osteoclasts can be generated *in vitro*, but osteoclast formation from human PBMCs typically takes 14–21 days [7-10]. Thus, if a putative resorption modulator is added to such cultures for a brief period, effects on resorption, measured as the area of bone surface excavated, will be observed against a high baseline of prior resorption; and if the modulator is added over a longer period, any change in resorption might be secondary to effects on differentiation.

This problem is partly circumvented in assays that measure bone resorption as the release into culture supernatants of products of bone solubilization [12,13,18]. This approach has the advantage that the quantity of solubilized product reflects only the quantity of bone resorbed since the last change of culture medium. This avoids the results being obscured by a baseline of prior resorption. However, even in such assays, bone resorption is measured over a period of 3 days, so that an unknown and potentially substantial component of any observed change in resorptive activity might have been due to an effect of the test agent on differentiation rather than function.

A second difficulty is the substantial inter-culture variability in osteoclast formation, which might be accentuated by the prolonged incubation required to form osteoclasts from PBMCs: long incubation times might magnify small initial differences between cultures, especially since cells capable of forming osteoclasts represent only a very small proportion (<1%) of PBMCs [15], so that random variation in this number could lead to appreciable inter-culture differences in osteoclast formation. High inter-culture variability requires more cultures per experimental group, and this restricts the number of groups that can be studied in each experiment.

We therefore developed a novel assay that, by addressing these difficulties, facilitates the measurement of osteoclast function independent of differentiation. First, we optimized bone resorption, since if there is more bone resorption it can be measured over a shorter period. Short measurement periods minimize the confounding effects of agents on differentiation. We optimized bone resorption by optimizing combinations of serum batches and PBMC-donors. This led to substantially greater and earlier release of CTX-I antigen, to levels an order of magnitude greater than previously reported [10,12,13]. This robust osteoclast formation enabled us to detect and measure CTX-I released in 1 h of incubation, a time sufficiently short that any change in resorption is likely to reflect an effect on osteoclastic function rather than on differentiation. Second, we minimized inter-culture variability by using each culture as its own baseline. Using this longitudinal approach, we found that changes in bone resorption could be detected using substantially fewer cultures per variable.

In view of the significant expense of longitudinal measurements of CTX-I, we tested whether measurement of the quantity of TRAP in the cell lysates at the termination of the experiment could substitute for the initial CTX-I assay. We found that, while TRAP correlated well with the initial CTX-I reading in control cultures, the correlation in experimental groups in which bone resorption was modulated was unreliable. We have previously noted that agents that modulate resorption also modulate lysate TRAP levels [16]. However, since TRAP release by resorbing osteoclasts is proportional to the duration of bone resorption, measurement of TRAP might more accurately reflect the osteoclast content of the culture in experiments in which resorption is measured over shorter time intervals.

## Conclusion

The short incubation times and the longitudinal sampling makes the assay we have described a powerful tool with which to detect and quantify the effects of agents that activate or modulate the resorptive activity of osteoclasts, while minimizing the potentially confounding effects of the same agent on osteoclastic differentiation. The assay design also reduces the inter-culture variability of resorption data, compared to previous approaches.

## Competing interests

UG and MS are employed by Medivir UK; BS is employed by Medivir AB. TJC receives consultancy fees from Medivir UK

## Authors' contributions

BK and KF performed all the experimental studies. KF prepared the figures. UG, BS, MS and TJC conceived the assay approach. UG, BS and MS created the novel inhibitors of cathepsin K. TJC supervised the experimental work and wrote the manuscript. All authors read and approved the final manuscript.
